# Studying histone inheritance in different systems using imaging-based methods and perspectives

**DOI:** 10.1042/BST20220983

**Published:** 2023-05-12

**Authors:** Emily Zion, Xin Chen

**Affiliations:** 1 Department of Biology, The Johns Hopkins University, 3400 N. Charles St., Baltimore, MD 21218, U.S.A.; 2 Howard Hughes Medical Institute, Department of Biology, The Johns Hopkins University, 3400 N. Charles St., Baltimore, MD 21218, U.S.A.

**Keywords:** epigenetics, histones, stem cells

## Abstract

Understanding cell identity is critically important in the fields of cell and developmental biology. During cell division, a mother cell duplicates the genetic material and cellular components to give rise to two daughter cells. While both cells receive the same genetic information, they can take on similar or different cell fates, resulting from a symmetric or asymmetric division. These fates can be modulated by epigenetic mechanisms that can alter gene expression without changing genetic information. Histone proteins, which wrap DNA into fundamental units of chromatin, are major carriers of epigenetic information and can directly influence gene expression and other cellular functions through their interactions with DNA. While it has been well studied how the genetic information is duplicated and segregated, how epigenetic information, such as histones, are inherited through cell division is still an area of investigation. Since canonical histone proteins are incorporated into chromatin during DNA replication and can be modified over time, it is important to study their inheritance within the context of the cell cycle. Here, we outline the biological basis of histone inheritance as well as the imaging-based experimental design that can be used to study this process. Furthermore, we discuss various studies that have investigated this phenomenon with the focus on asymmetrically dividing cells in different systems. This synopsis provides insight into histone inheritance within the context of the cell cycle, along with the technical methods and considerations that must be taken when studying this process *in vivo*.

## Introduction

A fundamental question in developmental biology is how cell identity is established and maintained. Cell identity is defined by the unique morphological, molecular, cellular, and functional characteristics that a cell possesses. Within a multicellular organism, hundreds of different cell types co-exist, all with distinct morphologies, molecular properties, cellular structures, and functional potentials. While the majority of cells within a multicellular organism carry the same genetic material, their unique characteristics could be influenced by chromatin structure, epigenetic mechanisms that help modulate cell-type-specific chromatin structure and gene expression programs without altering the DNA sequence [1], and other cellular components, such as regulatory RNAs, proteins, and organelles [2–4].

Cell fate decisions are challenged by cell division, when a mother cell divides to generate two daughter cells. Cell division modes can be either symmetric cell division (SCD), where the resulting daughter cells take on the same identity, or asymmetric cell division (ACD), where the daughter cells possess distinct cell identities [5,6]. In both scenarios, the genome must be faithfully duplicated and segregated. Therefore, the difference between these two modes could result from how epigenetic information is partitioned to regulate either identical or distinct daughter cell identities. While the duplication and segregation of genetic material has been well studied, the inheritance of epigenetic factors is a relatively new field with many remaining questions. Here, we will discuss the inheritance of histones as the main building blocks of chromatin and carriers of epigenetic information, as well as the methods and considerations that must be taken when studying this process *in vivo*. In particular, we will focus on discussing imaging-based studies during ACD in different systems.

## The biology of histone inheritance

To properly define cell identity, it is imperative that cell-type-specific genes are expressed while the remaining genes are silenced, which could be achieved through the modulation of chromatin states. The chromatin state at particular genomic regions can be more or less accessible and carry specific chromatin regulators, allowing regulation of gene expression [7]. Chromatin state can be profoundly influenced by histone and histone variant proteins, which directly interact with DNA. Four canonical histones (H2A, H2B, H3, and H4), form an octamer structure via an H3–H4 tetramer and two H2A–H2B dimers, and wrap ∼146–147 bp of DNA to form the basic unit of chromatin [8,9]. Histones serve as a major carrier of epigenetic information through their direct interaction with DNA for accessibility, as well as their numerous post-translational modifications [7,10].

**Figure 1.**
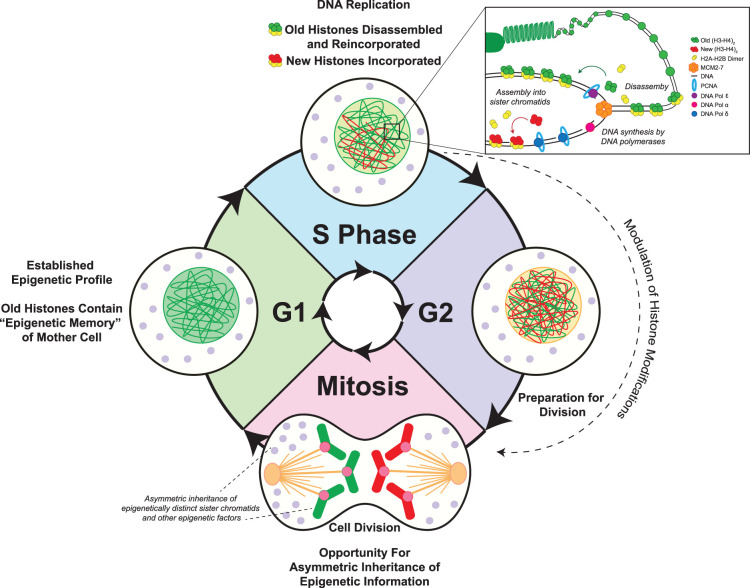
Epigenetic inheritance in the context of the cell cycle. Epigenetic inheritance occurs within the context of the cell cycle. In G1, the mother cell contains an epigenetic profile including specific histone modifications, chromatin states, and other epigenetic mechanisms that specify a unique cell identity. In preparation for mitosis, the mother cell enters S phase, where chromatin is disassembled to allow for DNA replication, and then reassembled into the resulting sister chromatids. This is an opportunity of maintenance or re-establishment of epigenetic information, as the preexisting or ‘old' histones that contain the epigenetic memory of the mother cell are reincorporated into chromatin, along with newly synthesized histones. For cells undergoing SCD, induced asymmetric histone inheritance could be detrimental even for cell fate change, such as differentiation of mouse embryonic stem cells [60]. The cell then enters G2 phase, where the sister chromatids can undergo further epigenetic changes, such as the addition of histone modifications and DNA methylation, known as chromatin maturation. In cells undergoing SCD, such as cultured mouse embryonic stem cells and yeast, a series of studies identified that the re-establishment of histone methyl marks requires both chromatin regulators and DNA sequences [61–63]. This process is important for cells undergoing SCD since both cells need to inherit similar epigenetic information, while certain histone modifications, such as methylations, are largely associated with old histones [20,64]. However, how this process is ongoing in cells undergoing ACD is still unclear, given the challenge that between the two daughter cells, epigenomic regions with commonality and difference are both present and likely regulated in sequence-specific manner, resulting in local epigenomic asymmetry [23,39,43]. From this point, the cell enters mitosis, where the mother cell divides into two daughter cells. This provides another opportunity for epigenetic information to be inherited symmetrically or asymmetrically through the inheritance of genetically identical while epigenetically similar or distinct sister chromatids, along with other cellular components, such as RNAs, proteins, and organelles. At many points throughout the cell cycle, epigenetic information can be maintained or changed to be inherited by the resulting daughter cells, which could be a fundamental feature for mitosis in multicellular organisms including mammals. Here, the establishment and segregation of globally asymmetric histone inheritance, such as in *Drosophila* ISCs and male GSCs, are depicted. Other situations are detailed in Figure 2.

During DNA replication, histones must be disassembled from chromatin to allow for the DNA polymerases to pass through. After replication, they must be reassembled into the two duplicated DNA strands to form sister chromatids, which are subsequently partitioned into two daughter cells during mitosis. These processes provide an opportunity to maintain or change the chromatin state, resulting in similar or different cell identities [11]. Since chromatin states are established prior to mitosis and partitioned during mitosis, it is important to understand histone inheritance patterns in the context of actively ongoing cell cycle [12] (Figure 1). The preexisting ‘old' histones that contain the information from the current cellular context can confer an ‘epigenetic memory'. In contrast, newly synthesized histones have not necessarily carried the same modifications as old histones and can establish a new chromatin state as well as gene expression program. Thus, it is important to understand how old and new histones are incorporated into chromatin during S phase, how their modification status may change during and after DNA replication in S-and-G2 phases, and how they are inherited by the resulting daughter cells through mitosis. Next, we will discuss techniques that can be utilized to interrogate different steps in establishing and partitioning of histones through the cell cycle.

## Methods to study histone inheritance

Both sequencing-based and imaging-based methods can be utilized to study histone inheritance. Sequencing-based methods can reveal genomic site-specific inheritance patterns but often lack spatial resolution and are commonly used with unicellular organisms such as yeast or cultured cells. Indeed, different genomics approaches have been developed and mainly applied on symmetrically dividing cells, which lead to much better understanding of the replication-dependent nucleosome reassembly [13–22]. Moreover, recently, genomics methods have been used to investigate transcriptome and epigenome changes in asymmetrically dividing cells in culture [23] or in multicellular organisms, such as during *Drosophila* embryogenesis [24]. In contrast, imaging-based methods are capable of visualizing histone inheritance patterns at single-cell resolution, but it is difficult to have the genomic sequence information at the same time. Future technology development is necessary to combine the strengths of both methods, in order to study histone inheritance with sequencing information at single-cell resolution *in vivo*. Here, we will focus on techniques used for imaging-based analysis of histone inheritance, complementary to many published reviews on sequencing-based methods to study this phenomenon [11,24–27].

### Histone labeling techniques

A straightforward method used to label a protein of interest is immunostaining, which uses a specific antibody to recognize a target antigen [28]. While this method can be specific, it requires sample fixation and permeabilization, raising the caveats of capturing snapshots that could lose the cell cycle context information without other labeling, as well as the long-standing issues concerning labeling efficiency and specificity of the antibody [29]. To combat this, nanobodies with more effective penetration and labeling have been developed [30]. These reagents can also be applied to live cells, further enhancing future antibody-dependent applications to label histones and histone modifications.

Additionally, tag-based techniques have been developed to label target proteins through genetic or chemical methods, generating a fusion protein with either a fluorescence or chemical tag. However, potential complications include changed function or localization of the tagged protein. It is prudent to ensure the tagged version of the target protein can substitute the endogenous version through experiments, such as rescuing loss-of-function mutants of the corresponding gene or co-localization shown by the antibodies against the endogenous protein. Expression of the fusion protein can be done using a transgene or by genome-editing to insert the tag at the endogenous gene locus [31]. The transgene method can provide spatial and temporal control, but the target protein is exogenously expressed, which could lead to effects and results hard to interpret [32]. Therefore, careful comparison is required to ensure exogenously expressed proteins recapitulate cellular features just like the endogenous proteins. On the other hand, tagging the endogenous gene allows for the labeled protein to be expressed under normal control, but this genome-editing could have off-targets and be technically challenging [31]. Additionally, for genes with multiple copies in the genome, such as histones, tagging one copy could lead to undetectable expression in the cells of interest [33].

More techniques have been developed that allow for labeling a protein of interest after it has been translated. For example, the SNAP-tag method utilizes SNAP enzymes that catalyze a covalent bond to ligands, such as a fluorescent dye [34]. Therefore, with SNAP-tagged protein expressed in specific cells, researchers can study differentially tagged proteins of interest with precise spatial and temporal control. While this method labels target proteins after being translated and can avoid issues such as RNA stability and protein perdurance, it brings back the concern of labeling efficiency as seen with the other methods discussed earlier. However, with the direct tagging techniques, the protein of interest could be labeled without the need for fixation or permeabilization of the cell. Therefore, these methods are more amenable to live cell imaging and could be used for sequentially labeling protein over long timescales.

### Distinguishing old and new histones

When studying epigenetic inheritance, an important concept is ‘epigenetic memory'. While certain epigenetic information must be inherited by both daughter cells to maintain their proper cellular memory, lack or change of symmetric inheritance could allow for a new cellular program. Thus, it is important to consider how epigenetic inheritance could occur in an equal or differential manner to influence daughter cell identities.

Histone proteins carry epigenetic information and can be post-translationally modified in many different ways. The differences in histone proteins (i.e. histones and histone variants), their genomic occupancies and densities, as well as their post-translational modifications could all affect their interaction with DNA, as well as how DNA interacts with other factors, to affect various biological processes [7,10]. Histones that are present prior to DNA replication, known as preexisting (‘old') histones, contain the epigenetic profile of the mother cell. Notably, many of the post-translational modifications on histones, such as methylation and phosphorylation, take time to accomplish [11]. During S-phase, new histones are synthesized to aid in the formation of chromatin with duplicated DNA. The newly synthesized histones are often lacking those modifications on old histone and can be thought of as a ‘blank slate', which could be modified in a similar or different manner. Considering these features, it is important to study how old and new histones are inherited during different modes of cell divisions. Here, we will discuss multiple methods for the labeling of histones and other epigenetic factors based on their ‘age' (i.e. synthesizing time) and corresponding biochemical properties, and considerations to take when studying epigenetic inheritance throughout the cell cycle.

A few techniques can differentially label old *versus* new histones in actively cycling cells, including the use of transgenes containing a genetic tag switch, photoconvertible fluorescent proteins, and covalent chemical pulse labeling. The initial studies of histone inheritance in stem cell ACD used the genetic tag switch method to label old and new histones [35–40]. In this design, a transgene driven by a tissue and cell type-specific promoter contains two fluorescent tagged histone coding sequences separated by a transcription termination site, which ensures the expression of these two tagged histones (e.g. GFP and RFP) is mutually exclusive. Upon a temporally controlled irreversible genetic switch, expression of one tagged histone will be terminated and expression of the other tagged histone will be turned on. In general, this tag switch method could be used to differentially label any protein of interest in a spatiotemporally controlled manner. When applying to histones, the precise distinguishment between the two populations of histones (i.e. old *versus* new) is only applicable in the context of the actively ongoing cell cycle, and this precision declines over time. For example, the later expressed tagged histones are new during the first S phase but will become old during subsequent S phases. Contrastingly, the earlier expressed tagged histones represent old histones more precisely, but the turnover of these histones could cause this signal to diminish in following cell cycles. Additionally, as this tag switch occurs at the DNA level, it will take time for the switch to be reflected at the protein level, considering RNA stability and protein perdurance of old histone, as well as the time needed for the new histone gene to be transcribed, translated, and properly localized. Thus, this method is more appropriate for cell types with relatively long cell cycles, and it is important to monitor the production and incorporation of new histone in a time-course experiment to understand the dynamics of the tag switch at the protein level in the context of ongoing cell cycles. Finally, as this method utilizes a transgene, the histones could be overexpressed, and it is important to ensure the overexpression has minimal to no cellular effects. When this method is properly utilized, it can provide great insights into histone inheritance patterns, as it is compatible with different imaging-based techniques, biochemical and molecular tools, as well as high-throughput sequencing-based epigenome profiling assays.

One caveat using this method is the delay for the tag switch from the DNA to the protein level. To circumvent this, other methods have the advantage to effectively change the histone protein features. One method, as discussed earlier, is the SNAP-tag method that utilizes an enzyme fused to the protein of interest that can covalently link to a fluorescent tag [34,41]. By introducing a pulse of the fluorescent tag substrate, the protein of interest can be labeled. Following a subsequent washout of the substrate and a chase, the newly synthesized proteins would be unlabeled. While this method could yield labeled protein faster than the genetic switch method, it is often used to label only one population of proteins. For example, in the regime discussed above, only old histones are positively labeled with a fluorophore, while new histones remain unlabeled. This could cause complications such as incomplete washout, particularly for cells in the tissue context, could result in imprecise labeling where both old and new histones are labeled. Moreover, since the new histones are unlabeled it could be difficult to identify them confidently without other markers for active cell cycle progression, such as nucleoside analog incorporation for completion of S phase. Theoretically, different labels could be introduced at different time points to label histone populations in a sequential manner using distinct pulse-chase and pulse regimes. Again, this would have to be done carefully in the context of the cell cycle, as defining the age of histones is only meaningful when considering active DNA replication.

Additionally, photoconvertible fluorescent proteins could provide an alternative label switching method that occurs at the protein level [42,43]. For example, the green fluorescent Dendra2 protein, after being exposed to blue light, can be irreversibly photoconverted to red fluorescent [42]. When using Dendra2-tagged histones in the context of the cell cycle, photoconverted red fluorescent histones represent old histones, while newly synthesized histones after the photoconversion would fluoresce green. Therefore, the two histone populations can be labeled with the temporal control of blue light exposure. Furthermore, when the Dendra2-tagged histones are expressed with tissue and cell-type specificity, spatial control could be achieved. One concern using this method is the photoconversion efficiency, since any unconverted old histones would fluoresce green and could be mistakenly analyzed as new histones. Additionally, this method requires cells of interest to be subjected to intense blue light exposure often executed in short and tandem pulses, which can cause DNA damage and other deleterious effects. Finally, since before and after photoconversion the protein stays the same regarding the primary sequences, any antibody-based epigenome assays will not be compatible with this method.

In summary, all three discussed methods here have advantages and disadvantages, and these methods have all been used to delineate histone inheritance patterns in different model systems (Table 1).

**Table 1:** Summary of methods to study histone inheritance

Method of analysis	Advantages	Disadvantages	Summary of literature findings
Sequencing-based analyses	Genomic, site-specific information of histone inheritance Used with unicellular organisms such as yeast or cultured cell lines	Lacks spatial resolution Not easily used with multicellular tissues with different cell types	Symmetric incorporation of H3 with slight bias to lagging strand in yeast [21] Symmetric incorporation of H3 with slight bias to leading strand in mouse ESCs [22]
Immunostaining	Single-cell resolution No need for genetic manipulations or protein tag No concerns about expression	Nonspecific labeling and labeling efficiency will influence results Labels all histones Requires fixation and permeabilization	Asymmetric segregation of histone variant H2A.Z, measured by antibody, in distributed stem cells (DSCs) [65,66]
Genetic Tag Switch	Labels two distinct populations of histones Can provide precise spatial and temporal control of tag expression and tag switch No need for fixation or permeabilization, amenable to live cell imaging	Exogenous expression, not under regulatory control Perdurance of tagged histone Longer time from genetic switch to protein readout	Global asymmetric H3, H4, CID inheritance in *Drosophila* male GSC and ISC ACD [35,37,38,40] Local H3 asymmetries in *Drosophila* female GSC ACD [39] Symmetric inheritance of H2A, H2B, H3.3 in *Drosophila* GSC and ISC ACD [35,38,40,59]
Photoconvertible Tag Switch	Protein-level tag switch that can identify two histone populations Quick time for tag switch Can provide spatial and temporal control No need for fixation or permeabilization, amenable to live cell imaging	Photoconversion must be done ex vivo Photoconversion efficiency must be measured Photoconversion can cause DNA damage and other deleterious effects	Local asymmetries of H3, H4 in mouse ESCs using Dendra2 photoconvertible protein [43]
SNAP-Tag System	Protein-level tag Different tags can be sequentially pulsed to label different populations Quick time to tag Can provide spatial and temporal control No need for fixation or permeabilization, amenable to live cell imaging	Inefficient washout can lead to misinterpretation of results Need to monitor efficiency of labeling One pulse only labels one population of histones Cell cycle context needs to be monitored to study labeled histone population	Symmetric inheritance of H3 and H3.3 parental histones in mouse muscle stem cells, occasional asymmetry [41]

### Considering the cell cycle progression

Epigenetic inheritance is a multi-step process that involves the establishment of heritable factors, as well as their organization and partitioning these factors through cell division (Figure 1). Thus, experimental design and interpretation must consider features at each distinct phases during cell cycle, in line with the timing and efficiency of the labeling or tag switch. For example, when studying the canonical histones whose incorporation into chromatin mainly occurs during DNA replication, it is necessary to design and perform experiments by timing the temporal labeling or tag switch with respect to the S phase. For old *versus* new canonical histone inheritance using the genetic switch method in asynchronous cells, the switch could occur at any time during the cell cycle but switched cell must be allowed to go through one complete S-phase for the new histones to be incorporated into the duplicated genome. If a label is added or the tag switch occurs after the completion of an S-phase, old and new histones are indistinguishable and studying their inheritance patterns in the upcoming mitosis would not be informative. If a label is added or the tag switch occurs during an S phase, there would be a mixture of new histones that are unlabeled and labeled, or differentially labeled, leading to caveats for data interpretation. Notably, the distinguishment between these two populations of histones is only precise for the first 1–2 cell cycles, as the newly labeled histones will become old and older in the subsequent cell cycles, as discussed above. On the other hand, to investigate old *versus* new histone variant inheritance, whose incorporation is not restrictive during DNA replication, such a requirement for a complete S phase will not apply. But full understanding of the cell cycle phase for the new histone variants to be incorporated is a prerequisite to design experiments and interpret results, as their incorporation modes are often cell type-specific and/or genome region-specific.

When distinct labels can reliably and differentially represent each population of histones, we would argue that the best timing during the cell cycle to tell their inheritance patterns is the anaphase or telophase during mitosis, as sister chromatids display clear separation at this moment while all subsequent cell cycle-dependent events have not occurred yet, such as the subsequent S phase-dependent new histone incorporation. For canonical histones, the subsequent G1 phase should still be ideal before the onset of S phase. Notably, many adult stem cells have a very short G1 phase and enter the subsequent S phase almost immediately after M phase [44,45], restricting the usage of G1 phase to analyze histone inheritance patterns. Contrastingly, for histone variants, more caution needs to be taken as it has been reported that certain histone variant, such as the centromere-specific H3 variant CENP-A, could be incorporated at anaphase [46]. Additional considerations should be taken, for example, if immunostaining strategy is applied, the mitotic chromosomes are condensed and often inaccessible to antibodies. Therefore, using fluorescent labels without the need to stain anaphase and telophase chromosomes would give out more confident results. In summary, rigorous criteria must be considered and applied to ensure a designated and recognizable feature accurately reflect the behavior of a particular histone population in the context of an actively progressing cell cycle.

## Current studies of histone inheritance in different systems

The labeling methods discussed here, along with cell cycle information, can be used with high-throughput sequencing methods, proteomics approaches, and imaging-based analyses to study how epigenetic factors, such as histones, are inherited through mitosis. The current studies of histone inheritance utilize a combination of these methods in different systems to understand how histones are inherited and how distinct inheritance patterns influence cell fate decisions. While significant advances have been made in understanding histones and histone modifications in regulating gene expression, our knowledge of how this information is maintained or changed during cell divisions, especially in multicellular organisms, is lacking.

**Figure 2.**
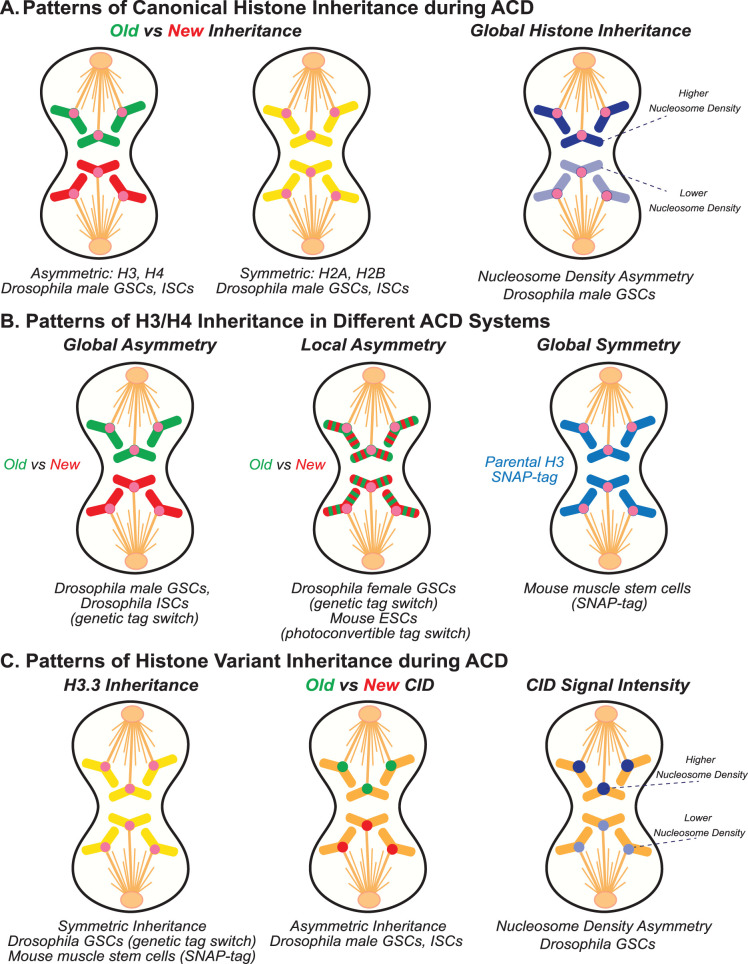
Different histone inheritance patterns in different systems. A variety of histone inheritance patterns detected in different stem cell systems. (**A**) General patterns of canonical histone inheritance during ACD. When studying old *versus* new histones, two general patterns have been observed, asymmetric inheritance of these two populations as seen with H3 and H4, and symmetric inheritance of these two populations as seen with H2A and H2B. These observations have been made in the asymmetrically dividing *Drosophila* ISCs and male GSCs. Asymmetric inheritance of nucleosomes has also been observed in *Drosophila* male GSCs, with the self-renewed GSC inheriting higher nucleosome density than the differentiating daughter cell. (**B**) A variety of H3/H4 inheritance patterns have been detected in different ACD systems. Global asymmetry of H3 and H4 have been identified using the genetic tag switch method in *Drosophila* ISCs and male GSCs. Furthermore, local H3/H4 asymmetries have been reported using the genetic tag switch in *Drosophila* female GSCs, as well as in mouse ESCs using a Dendra2 photoconvertible tag. Finally, globally symmetric inheritance of parental H3 has been observed in mouse muscle stem cells using the SNAP-tag method. (**C**) Inheritance patterns of histone variants during ACD have also been reported. In *Drosophila* GSCs, old and new H3.3, a variant of H3, is inherited symmetrically during ACD using the genetic tag switch. Furthermore, parental H3.3, labeled by a SNAP-tag, has been shown to be inherited symmetrically in mouse muscle stem cells. Old and new CID, the centromeric variant of H3, has been shown to be inherited asymmetrically in *Drosophila* male GSCs and ISCs. Additionally, asymmetric inheritance of total CID nucleosome density has been reported in *Drosophila* male and female GSCs.

To understand histone inheritance during ACD, a pivotal study was conducted in the *Drosophila* male germline stem cell (GSC), which undergoes ACD to produce a self-renewed GSC and a differentiating daughter cell [35]. Using a genetic tag switch approach to differentially label old *versus* new histones, global asymmetric inheritance of histones H3 and H4 was identified, where old H3/H4 were preferentially retained by the self-renewed GSC and new H3/H4 were enriched towards the differentiating cell [35,38] (Figure 2A,B). This was in contrast with the inheritance of old and new H2A/H2B, which showed globally symmetric inheritance between the two daughter cells [38,40] (Figure 2A). This distinction could be due to the different modes that old H3 and H4 are reincorporated as a tetramer, while old H2A and H2B split into two dimers following their dissociation from DNA strands during replication [47–51]. Moreover, H3 and H4 carry most of the post-translational modifications that influence gene expression [7,52–54]. This suggests that old histones H3 and H4 may retain an ‘epigenetic memory' to maintain the ‘stemness' of the stem daughter cell while the new histones serve to reset the chromatin of the differentiating daughter cell. Furthermore, this asymmetric inheritance was shown to be important for proper daughter cell identity, as when this inheritance was perturbed using a dominant histone H3 mutant that mutates the Thr3 to Ala (H3T3A), phenotypes such as germ cell loss and germ cell tumors were detected. Similar phenomena have also been reported in the ACD of *Drosophila* intestinal stem cells (ISCs), where old H3/H4 are preferentially retained by the self-renewed ISC and disruption of this asymmetry results in accumulation of ISC-like cells [40] (Figure 2A,B).

In *Drosophila* female GSCs, old and new H3 and H4 show localized asymmetries at genomic regions harboring either stemness or differentiation genes, suggesting a potential role in regulating differential gene expression between the daughter cells [39] (Figure 2B). Furthermore, using photoconvertible Dendra2-tagged histones in induced asymmetrically dividing mouse embryonic stem cells (mESCs), similar local asymmetries of old and new H3/H4 have been shown [43] (Figure 2B). Using single-cell transcriptome and epigenome co-profiling, differences in the H3K27me3 but not the H3K4me3 histone modification have been identified at differentially expressed genes between the resulting daughter cells [23]. These studies demonstrate that both global and local asymmetric histone inheritance could be detected in different systems. It will be interesting to explore whether these differences support the adoption of distinct gene expression programs or other biological readouts in the resulting daughter cells.

The inheritance of H3 and the H3.3 variant have also been studied in mouse muscle stem cells after injury using the SNAP-tag method [41]. Here, generally symmetric inheritance of H3 and H3.3 have been reported in both ACD and SCD of these stem cells, with a small population showing asymmetric H3 inheritance (Figure 2B,C). Notably, these experiments were designed with one pulse of SNAP labeling to tag old H3, with new H3 remaining untagged. Without other markers to ensure that the analyzed cells have undergone a complete S-phase, it would be difficult to pinpoint how specific the labeling is for old histones. In addition, with the untagged new histones it would be challenging to analyze any local histone asymmetries.

In addition to studying the inheritance patterns of canonical histones regarding their age (i.e. old *versus* new) and the distinct post-translational modifications they carry, there are a multitude of histone-related avenues that could influence molecular and cellular features in the resulting daughter cells. For example, nucleosome density and composition (e.g. with canonical histones *versus* histone variants) have profound impacts on the accessibility and structure of chromatin. A recent study demonstrated asymmetric nucleosome density on sister chromatids inherited by the daughter cells during the ACD of *Drosophila* male GSCs [55] (Figure 2A). This density asymmetry resulted in asynchronous cell cycle progression, where the differentiating daughter cell that inherited the set of sister chromatids with lower nucleosome density enters the subsequent S-phase prior to the self-renewed stem daughter cell. Furthermore, most of the studies focused on canonical histones that are mainly incorporated into chromatin in a replication-dependent manner. Histone variants are structurally and functionally distinct from the canonical histones, and their incorporation does not depend on DNA replication. In *Drosophila* ISCs, the centromeric H3 variant, CID (in flies) or CENP-A (in mammals), has been found to be asymmetrically inherited based on age, with the old CID preferentially retained by the ISC during ACD [37] (Figure 2C). Moreover, asymmetric amount of CID has been shown to be inherited in both male and female GSCs of *Drosophila*, with the self-renewed stem cells inheriting more CID than the differentiating daughter cells [56–58]. Because sister chromatids have the same DNA sequences, this amount difference reflects the CID-containing nucleosome density difference between sister centromeres (Figure 2C). Another H3 variant, H3.3, which is incorporated into chromatin in a transcription-dependent manner, segregates symmetrically at a global level in *Drosophila* male GSCs based on their age (i.e. old *versus* new H3.3) [35,59] (Figure 2C). It remains to be addressed whether there is any local asymmetry of old *versus* new H3.3 and whether there is any H3.3-containing nucleosome density difference between sister chromatids. A local H3.3 asymmetry could correlate with and/or contribute to distinct local gene expression activities, an interesting possibility awaiting further studies.

## Perspectives

- Current research that uses labeling techniques to identify old and new histones has identified unique inheritance patterns of histones in different systems, including both global and local asymmetric inheritance of old and new histones, as well as asymmetric nucleosome density.
- Future investigations are necessary to understand the generality of this phenomenon, as well as the downstream biological effects of different histone inheritance patterns. Technology development to allow visualization of both epigenetic and genetic information simultaneously at single-cell resolution will greatly facilitate similar studies *in vivo*.
- Careful analysis of histone behaviors will give greater insight into histone inheritance patterns and biological significance in a wide variety of systems, including different adult stem cell systems in homeostasis and tissue regeneration, as well as cell divisions leading to major lineage specifications during development of multicellular organisms. With this knowledge, we will be in a better position to understand how mis-regulation of these processes could lead to diseases such as cancers and tissue degeneration, as well as developmental defects, many of which lack known genetic mutations.

## Abbreviations

ACD: asymmetric cell division
GSC: germline stem cell
ISCs: intestinal stem cells
SCD: symmetric cell division
